# A conceptual framework for rationalized and standardized Universal Newborn Hearing Screening (UNHS) programs

**DOI:** 10.1186/s13052-016-0223-1

**Published:** 2016-02-12

**Authors:** Carlo Giacomo Leo, Pierpaolo Mincarone, Saverio Sabina, Giuseppe Latini, John B. Wong

**Affiliations:** National Research Council of Italy, Institute of Clinical Physiology, Unit of Lecce (CNR-IFC), c/o Campus Universitario Ecotekne, Via per Monteroni, Lecce, 73100 Italy; Tufts Medical Center, Department of Medicine, Division of Clinical Decision Making, 800 Washington St, Boston, MA 02111 USA; National Research Council of Italy, Institute for Research on Population and Social Policies (CNR-IRPPS), Research Unit of Brindisi, c/o ex Osp. Di Summa, Central Building Floor 1 Office 18 - P.zza Di Summa, Brindisi, 72100 Italy; Tufts University, School of Medicine, 145 Harrison Avenue, Boston, MA 02111 USA

**Keywords:** Universal Newborn Hearing Screening, Checklist, Quality improvement, UNHS

## Abstract

Congenital hearing loss is the most frequent birth defect. The American Academy of Pediatrics and the Joint Committee on Infant Hearing established quality of care process indicators for Universal Newborn Hearing Screening starting from 1999. In a previous systematic review of Universal Newborn Hearing Screening studies we highlighted substantial variability in program design and in reported performance data. In order to overcome these heterogeneous findings we think it is necessary to optimize the implementation of Universal Newborn Hearing Screening programs with an appropriate application of the planning, executing, and monitoring, verifications and reporting phases. For this reason we propose a conceptual framework that logically integrates these three phases and, consequently, a tool (a check-list) for their rationalization and standardization.

Our paper intends to stimulate debate on how to ameliorate the routine application of high quality Universal Newborn Hearing Screening programs. The conceptual framework is proposed to optimize, rationalise and standardise their implementation. The checklist is intended to allow an inter-program comparison by removing heterogeneity in processes description and assessment.

**Electronic supplementary material:**

The online version of this article (doi:10.1186/s13052-016-0223-1) contains supplementary material, which is available to authorized users.

## Background

Sensorineural hearing loss is one of the most frequently occurring permanent congenital defects at birth with a prevalence of 0.1–0.3 % for newborns [1–4] (2–5 % in presence of audiological risk factors) [5]. Its late diagnosis could negative influence language, learning and speech development with lifelong consequences [6–11]. Universal Neonatal Hearing Screening (UNHS) programs were developed in several countries to identify the majority of newborns with hearing impairment. UNHS programs adopt, as screening tests, otoacoustic emissions (OAEs) and/or automated auditory brainstem response (aABR) testing. Those who are positive at tests are referred to full audiological diagnosis. Audiological or medical/surgical management, educational and (re)habilitation methods, and child and family support are available strategies for subjects with confirmed hearing loss [12]. Recognised benefits of UNHS are better language outcomes at school age and improved long-term language development [13, 14].

Since the 1999, process and outcome performance indicators and benchmarks were established for Early Hearing Detection and Intervention (EHDI) programs (i.e., identification before 3 months of age and intervention by 6 months of age) [15] to evaluate progress and determine consistency and stability [16, 17]. In 2007 the JCIH recommended timely and accurate monitoring of relevant quality measures, based on its reviewed performance indicators and benchmarks, as an essential practice for inter-program comparison and continuous quality improvement [17].

With the aim to verify whether literature reporting experiences on hospital-based UNHS programs include sufficient information to allow inter-program comparisons according to the already available indicators/benchmarks defined by the AAP and JCIH, we performed a systematic review [18]. We found that not all studies reported all the data necessary for calculating the complete proposed set of quality indicators, and that when comparing available data on indicators with corresponding benchmarks, the full achievement of all the recommended targets is an open challenge. We also found substantial heterogeneity in terms of extent of hearing loss (hearing threshold, uni- vs. bilateral hearing loss), criteria for identification of neonates at higher risk of hearing loss, screening tests used, personnel performing the tests, testing environment.

In order to overcome these heterogeneous findings we think it is necessary to optimize the implementation of Universal Newborn Hearing Screening programs with an appropriate application of the planning, executing, and monitoring, verifications and reporting phases. For this reason we propose a conceptual framework that logically integrates these three phases and, consequently, a tool (a check-list) for their rationalization and standardization.

## Discussion - The conceptual framework for rationalized and standardized UNHS programs

**Fig. 1:**
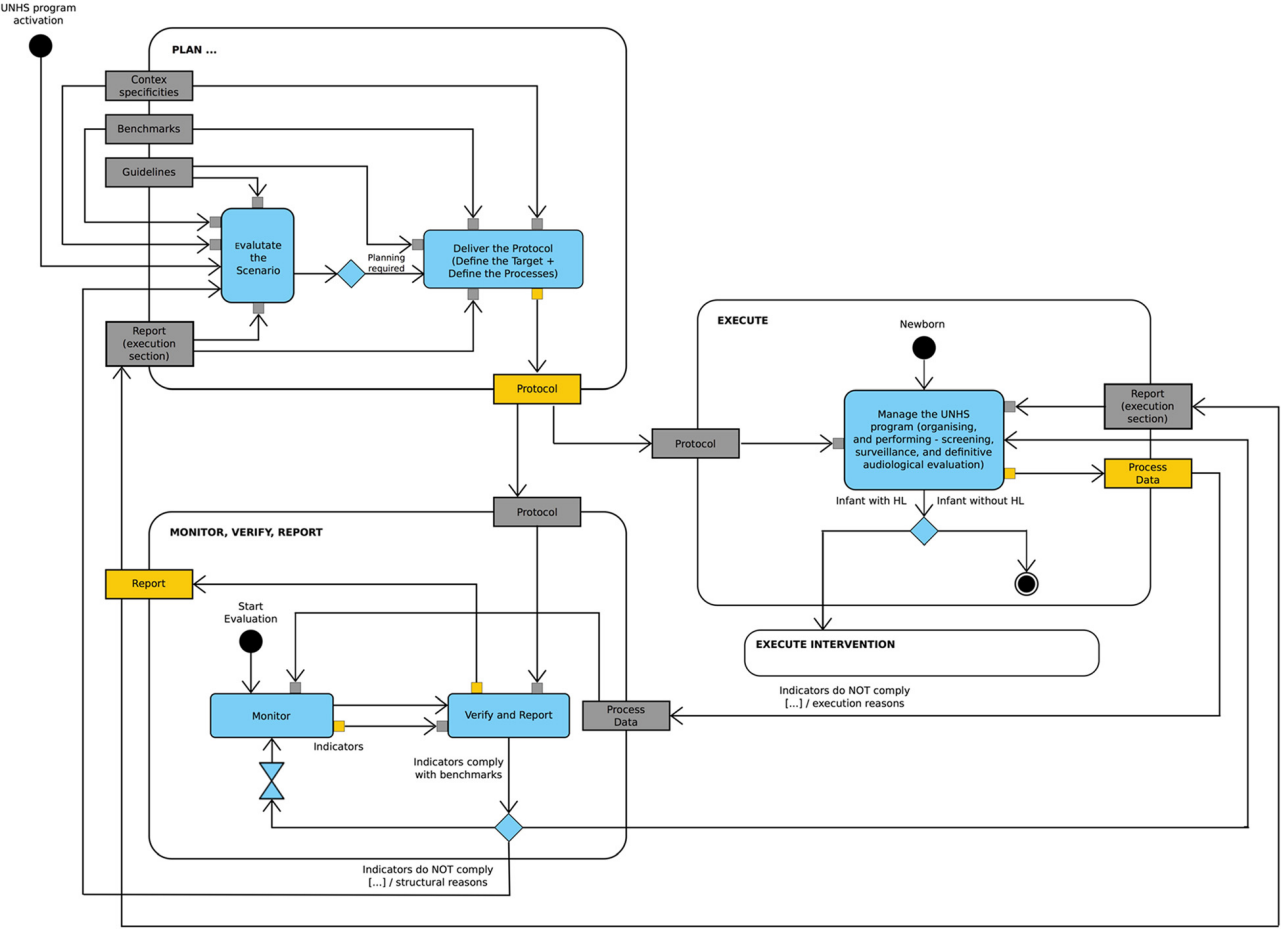
A framework for managing a UNHS program – general view. The framework is composed by three phases: a planning phase based on indications from guidelines and recommendations, specificities of the local context, benchmarks, reports from verification phase; an executing phase where the protocol is applied and where data are generated, managed and aggregated for monitoring; a monitoring, reporting and verifying phase where the indicators are compared with benchmarks and a report is generated. The Unified Modeling Language™ (UML®) notation is adopted for representation purposes: black filled circle represents the initial state and rounded circle the ending state; the rounded square indicates an action and the diamond a gateway where the process can take different roots upon a specified conditions; the arrowed line depicts the flow and the stylized sand-glass a time event (specifically adopted for a waiting time); the sharp-cornered square is used for objects/data/information

**Fig. 2:**
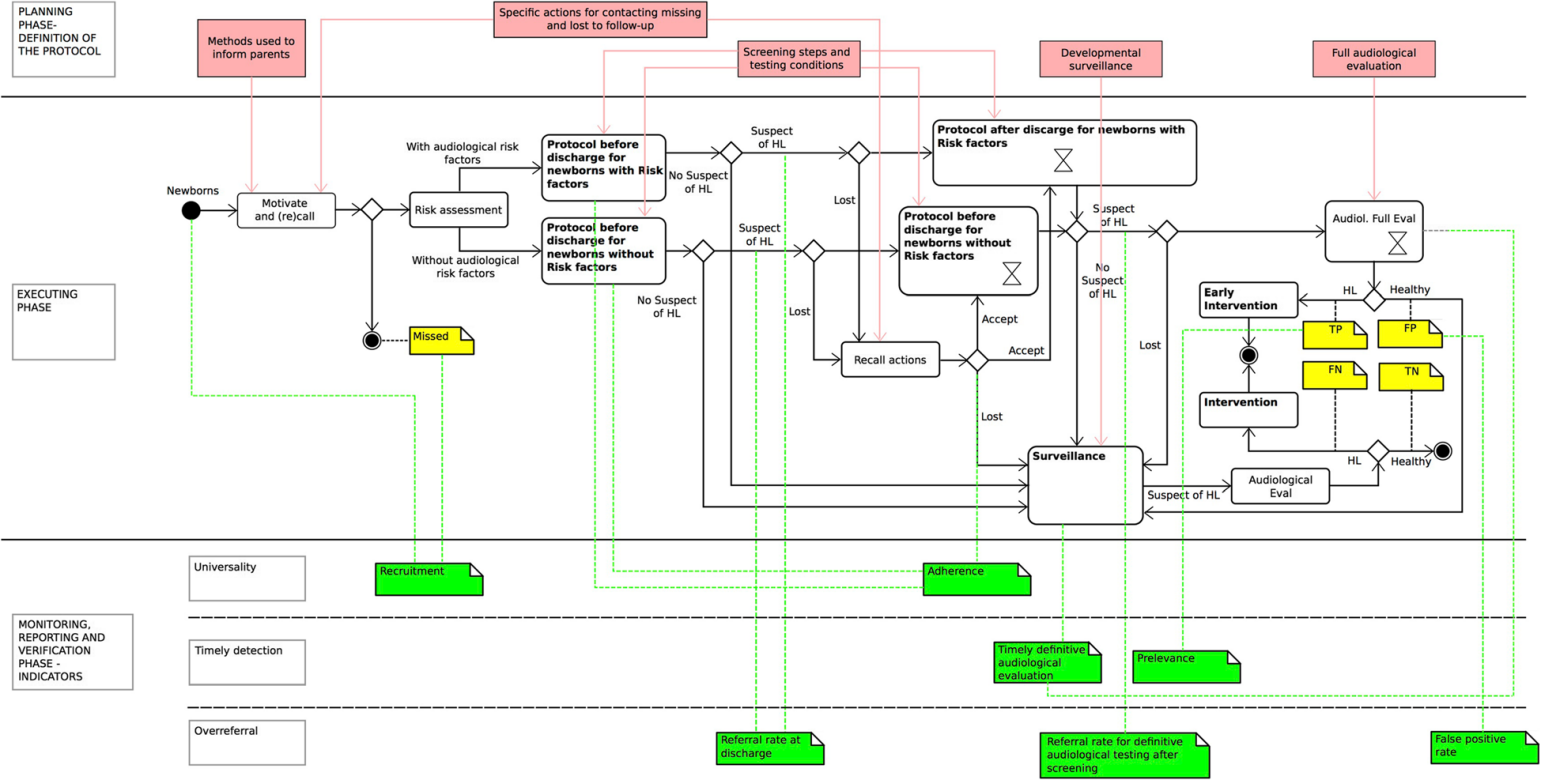
A framework for managing a UNHS program – detailed description. A detailed description of the conceptual framework is provided. In the central lane a description of the executing phase is reported recurring to the UML® notation: in addition to the specifications in Fig. 1, the folded square is used for noting. The key elements of the planning phase are reported on the top of the Figure and the proposed indicators on the bottom. In both cases their impact on the UNHS process is represented recurring to vertical lines ending on a specific point in the executing phase

The framework is structured on several phases (see also Fig. 1 and Fig. 2):

1. A planning phase based on indications from guidelines and recommendations, specificities of the local context, benchmarks, reports from verification phase. The *Deliver the protocol* Action is activated, apart from the first instance, if the benchmarks are not achieved, if guidelines/recommendations are updated, or if the context specificities change. The output of this phase is the protocol for UNHS execution.
2. An executing phase where the protocol is applied and where data should be generated and managed for monitoring. The outputs of this phase are the raw data for process monitoring.
3. A monitoring, reporting and verifying phase where the *Monitor* action is activated periodically or upon request to aggregate data and build performance indicators. The verification is made comparing indicators with benchmarks and a report is generated with an analysis of reasons for possible deviations which can push for a redefinition of the protocol and/or for a re-organization of its execution. Reports can be used also for disseminating purposes.

A more detailed description of the single phases follows in the following sections (note that the *EXECUTING UNHS PROGRAM* phase is out of the scope of our work and will not be discussed further).

### Planning UNHS program

In order to deliver a protocol it necessary to define the target and the processes.

#### Definition of the target

Two elements are of importance: the definition of hearing loss and the identification of criteria used to define newborns at higher risk of hearing loss. *Hearing threshold* and uni- vs. bilateral have an impact in the number of neonates going through testing and evaluations, the number of infants admitted to therapy, the rate of newborns with hearing loss with early diagnosis and treatment, the number of neonates that could erroneously be evaluated as with no hearing deficits. These two parameters are fundamental for inter-program comparison. Several classifications of Hearing Loss have been formulated [19–21] which brings in the definition of the levels of severity (see Additional file 1). It is therefore necessary to make the choice explicit also for their impact on the typology of treatment/rehabilitation. We have previously observed that, in the lack of standardization, several thresholds have been applied in UNHS programs (26 to 40 dB HL).(6) *Newborns with risk factors* for neonatal hearing loss have about a 10 fold probability for hearing deficits with respect to the overall population [1, 2]. Criteria for higher audiological risk have been defined by several subjects (JCIH [17], the US National Institutes for Health - NIH [22], ASHA [23]) or are chosen directly by program coordinators (e.g., Clemens et al. [24]).

The audiological risk criteria are relevant for that: specific audiological risk may require a specific screening protocol; the timing and number of hearing re-evaluation (surveillance) for infants at risk should be customized and individualized depending on the relative likelihood of a subsequent delayed onset hearing loss.

#### Definition of the processes

Activities, detailed actions, decision nodes, workflows, roles, environmental conditions have to be identified and specified. More specifically key issues are reported. *The typology and the number of tests, and the healthcare setting in which performing the examinations* (before or after the discharge) – The program needs to be well balanced for sensitivity, specificity, coverage of the population and costs per subject identified. E.g., Kennedy et al. [25] reported to have changed their protocol using unilateral failure on aABR, rather than bilateral failure on Transient Evoked OAEs (TEOAEs) testing, as the second step; this change was associated with a reduction in the screen-failure rate from 2.4 % (95 % CI 2.2–2.6) to 1.3 % (1.1–1.5) of babies screened. *The presence of specific protocol for neonates at higher risk* (e.g., aABR for NICU staying in NICUs for more than 5 days instead of TEOAEs) [17] - Such neonates, in fact, are at risk of having neural hearing loss (auditory neuropathy/auditory dyssynchrony) which is not detectable with TEOAEs*. The set of examinations for the full audiological evaluation* – E.g., the one recommended by the JCIH [17]. *The tasks to be performed to increase the percentage of enrolment and to reduce the lost to follow up* (neonates referred to further examinations that do not show at the planned appointments) - With reference to the former, it has to be noted that specific actions should be done for an appropriate communication with families creating the conditions for an informed consent. With reference to the latter, a survey conducted in USA [26] shows that only 62 % of all newborns who need a diagnostic evaluation actually did it and, out of them, only 52 % by the age of 3 months (as recommended by the JCIH). The lost to follow-up at all stages of the EHDI process continues to be a serious concern also for the World Health Organization (WHO) [27] that states the importance, for its success, of monitoring and implementing all the phases of the screening (responsibilities, training, information campaign, procedures of quality assurance). *The surveillance program for early identification of infants and children with late onset* (especially in presence of high risk factors) – It is recommended to perform regular surveillance of developmental milestones, auditory skills, parental concerns, and middle-ear status to for all infants, together with an objective standardized screening of global development, at 9, 18, and 24 to 30 months of age or at any time if the health care professional or family has concern [17]. *The cooperation among all the involved operators, services and institutions* - The identification of the key roles is an essential step for an appropriate management of the entire process and for monitoring purposes.

### Monitoring, verifying and reporting

#### Monitoring

In our systematic review [18] the AAP [16] and JCIH [17] performance indicators focused on early diagnosis of neonates with hearing loss have been presented in detail and grouped in three areas:

1. *Universality*– completeness of universality in both recruitment and follow-up phases;
2. *Timely detection*– specification of follow-up deadlines for identification and intervention, and determination of the observed prevalence;
3. *Overreferral* – Efficient use of highly specialized care.

#### Verification of program performance

For each reported indicator a reference benchmark has been identified [18] which represent a consensus of expert opinion in the field of newborn hearing screening and intervention, and are the minimal requirements that should be attained by high quality EHDI programs. Frequent measures of quality permit prompt recognition and correction of any unstable component of the EHDI process. The JCIH recommends timely and accurate monitoring of relevant quality measures for inter-program comparison and continuous quality improvement [17].

#### Reporting of process indicators

This is the output of the *Monitor, Verify and Report* Activity and it is used to make process results explicit and, as previously reported, as the basis for possible re-planning and/or reorganization.

The proposed framework has been used as a conceptual guidance for building a checklist (Additional file 2) intended to support UNHS program coordinators in the planning, monitoring, reporting and verification.

## Conclusions

As reported in the 2007 JCIH Position Statement [17], regular measurement of performance and routine monitoring of indicators are recommended for inter-programme comparison and continuous quality improvement. With the aim of achieving high quality UNHS programs by achieving AAP and JCIH quality benchmarks, our work proposes a conceptual framework and a checklist. The former is a way to optimize, rationalise and standardise the implementation of UNHS programs by considering all the relevant phases: Planning, executing, and monitoring, verifying and reporting. The latter allows an inter-program comparison by removing heterogeneity in processes description and assessment.

The paper is a contribution toward a standardisation in reporting UNHS experiences which may favour the emerging of best practises.

## Additional files

Additional file 1:
**The need of an explanation: what is the target Hearing Loss for UNHS?.** (PDF 82 kb) Additional file 2:
**Check list for the quality of Universal Newborn Hearing Screening programs.** (DOC 94 kb)

## Abbreviations

aABR: Automatic auditory brainstem response
AAP: American Academy of Pediatrics
EHDI: Early hearing detection and intervention
JCIH: Joint Committee on infant hearing
NICU: Neonatal intensive care unit
OAEs: Evoked otoacoustic emissions
TEOAEs: Transiently evoked OAEs
UNHS: Universal Newborn Hearing Screening
