# Supplementary material for: A conceptual framework for rationalized and standardized Universal Newborn Hearing Screening (UNHS) programs
Source: Ital J Pediatr. 2016 Feb 12;42:15. doi: 10.1186/s13052-016-0223-1 (PMC4751642; doi:10.1186/s13052-016-0223-1)
Supplement: Additional file 1: — The need of an explanation: what is the target Hearing Loss for UNHS?. (PDF 82 kb) [file 13052_2016_223_MOESM1_ESM.pdf]

Additional File 1

The need of an explanation: what is the target Hearing Loss for UNHS?

Before starting with a universal newborn hearing screening, it is necessary to answer this question: what does hearing loss mean? According to Nelson and collegues[1] the targeted hearing loss for UNHS programs is permanent sensory or conductive hearing loss averaging 30 to 40 decibels (dB) or more in the frequency region important for speech recognition (approximately 500 through 4000 Hertz [Hz]). The focus of UNHS is on congenital as opposed to acquired or progressive hearing loss that may not be detected in the newborn period." This answer implies that, having the threshold for "normality", it is possible to define the different levels of hearing loss as *mild*, *moderate*, *severe* or *profound*. However, in our review[2] we have observed that, in the lack of standardization, not only there are different thresholds of hearing loss (from 26 to 40dB), but also that there are different ways to classify its severity. If the first difference can be justified by the adherence to the "normal" threshold or by considering the particular condition that characterizes the hearing screening of the newborns (presence of amniotic fluid or vernix in the auditory canals which can affect TEOAE examinations) to reduce the false positives, the same explanation cannot be used for the different values used to classify the severity. In fact, if, for example, we compare the grade of hearing impairment of the World Health Organization (WHO),[3] of the International Bureau for Audiophonology (BIAP)[4] and of the American Speech-Language-Hearing Association (ASHA)[5], we find differences in the description of the severity and in the average hearing level (Table 1). We need standardization or evidence by which comparing the different protocols.

Table 1: Description of the severity and average hearing level (value are expressed in dB) in three different Institutions (WHO, BIAP, ASHA)

**Description of the severity**	**Average hearing level** (value are expressed in dB)		
	**WHO**	**BIAP**	**ASHA**
Normal	25 or less	20 or less	10 - 15
Slight	26 - 40	N.R.	16 - 25
Mild	N.R.	21 - 40	26 - 40
Moderate	Child 31 - 60<br>Adult 41 - 60	1st degree 41 - 55<br>2nd degree 56 - 70	41 - 55
Moderately severe	N.R.	N.R.	56 - 70
Severe	61 - 80	1st degree 71 - 80<br>2nd degree 81 - 90	71 - 90
Profound	81 +	Very severe<br>1st degree 91 - 100<br>2nd degree 101 - 110<br>3rd degree 111 - 119	91 +
Total hearing loss	N.R.	120 +	N.R.

References

1. Nelson HD, Bougatsos C, Nygren P. Universal newborn hearing screening: systematic review to update the 2001 US Preventive Services Task Force Recommendation. Pediatrics. 2008;122(1):e266-76. doi:10.1542/peds.2007-1422.
2. Mincarone P, Leo CG, Sabina S, Costantini D, Cozzolino F, Wong JB et al. Evaluating reporting and process quality of publications on UNHS: a systematic review of programmes. BMC Pediatrics. 2015;15(1):86-. doi:10.1186/s12887-015-0404-x.
3. World Health Organization. Primary Ear And Hearing Care Training Resource. Advanced Level., Geneva. 2006. https://www.entnet.org/sites/default/files/Community/public/upload/WHO-Advanced-Ear-Care.pdf. Accessed 11/07/2014.
4. Bureau International d'Audiophonologie. BIAP Recommendation 02/1 bis. 1996. http://www.biap.org/index.php?option=com_content&view=article&id=5%3Arecommandation-biap-021-bis&catid=65%3Act-2-classification-des-surdites&Itemid=19&lang=en. Accessed 11/07/2014.
5. Clark JG. Uses and abuses of hearing loss classification. Asha. 1981;23(7):493-500.
