# Supplementary material for: A conceptual framework for rationalized and standardized Universal Newborn Hearing Screening (UNHS) programs
Source: Ital J Pediatr. 2016 Feb 12;42:15. doi: 10.1186/s13052-016-0223-1 (PMC4751642; doi:10.1186/s13052-016-0223-1)
Supplement: Additional file 2: — Check list for the quality of Universal Newborn Hearing Screening programs. (DOC 94 kb) [file 13052_2016_223_MOESM2_ESM.doc]

**Table 1:**

**Check list for the quality of Universal Newborn Hearing Screening programs**

**PLANNING PHASE - TARGET AND PROTOCOL DESCRIPTION**

DEFINITION OF THE TARGET

- Classification of severity of HL based on (see Table 1 of Additional file 1 for details):
  - *Primary Ear And Hearing Care Training Resource. Advanced Level. Geneva: WHO; 2006;[1]*
  - *BIAP Recommendation 02/1 bis; 1996;[2]*
  - *Clark JG. Uses and abuses of hearing loss classification. ASHA. 1981 Jul;23(7):493-500;*
  - *Other, specify either*
    - - Source: ___________________________________________

________________________________________________ or

- - - - Details

| **Hearing impairment level[[1]](#footnote-2)** | **Hearing levels dB HL** |
| --- | --- |
| Normal Hearing | 0 - … |
| ___________ | _____ |
| ___________ | _____ |
| ___________ | _____ |
| ___________ | _____ |
| ___________ | _____ |
| ___________ | _____ |

- Uni- vs. Bilateral hearing loss:
  - *Unilateral*
  - *Bilateral*
- Criteria used for the evaluation of audiological risks:
  - JCIH, 2007
  - Admission to NICU for at least ____ days
  - Other ________________________________________________________

DEFINITION OF THE PROTOCOL

- Screening Steps for neonates with and without audiological risk factors

| **Step n.** | **Protocol for general population** | | **Protocol for special cases (as per previous point)** | |
| --- | --- | --- | --- | --- |
| 1 | Test^a^______________  Age^b^ ______________ |  | Test^a^______________  Age^b^______________ |  |
|  |  | After discharge |  | After discharge |
| 2 | Test^a^______________  Age^b_^ _____________ | ☐ | Test^a^______________  Age^b^ ______________ | ☐ |
| 3 | Test^a^______________  Age^b^ ______________ | ☐ | Test^a^______________  Age^b^ ______________ | ☐ |
| 4 | Test^a^______________  Age^b^ ______________ | ☐ | Test^a^______________  Age^b^ ______________ | ☐ |

^a^ e.g. TOAE, aABR or other

^b^ correct age for preterm

- Testing environment conditions (more than one choice allowed)
  - NICU
  - Silent room [report the acceptable noise level in dB _______________]
  - Mother’s bed
  - Other_________________________
- Full audiological evaluation (check all the applied tests)
- Otoscopic inspection,
- Child and family history,
- Impedance,
- OAE
- ABR
- Frequency-specific tone burst
- Air and bone conduction
- Sedation capability
- _________________
- Methods used to inform parents about the importance of screening, procedures, costs, potential risks of hearing loss, and the benefits of early detection and intervention (check all that apply)
  - Discussion with specialized personnel
  - Flyers/Posters
  - Other____________________
- Specific actions to contact parents of newborns who missed a step or were lost to follow-up (check all that apply)
  - Telephone call
  - Letter from hospital
  - Contact pediatrician
  - Other _________________________
  - None
- Developmental surveillance:
  - Used Tests[3]: ___________________________; Timing: ___________________________
  - (in alternative) Free description: ______________________________________
  - Potential false negatives (neonates with hearing loss but negative to previous screening tests) are detected. In case, the following method is used
  - Follow-up with pediatricians
  - Follow-up with services responsible for assigning hearing aids
  - Recall of all the investigated neonates after a certain period of time
  - Other ____________________________
- Professionals involved in each screening step (excluding the final audiological evaluation)

|  |  | **Personnel** | | | |
| --- | --- | --- | --- | --- | --- |
| **Role** | **Physician** | **Trained nurse** | **Audiologist** | **Family pediatrician** | **Other** |
| Screening program responsible | □ | □ | □ | □ | □ |
| Communication with parents | □ | □ | □ | □ | □ |
| Evaluation of risk factors | □ | □ | □ | □ | □ |
| Neonate wellness | □ | □ | □ | □ | □ |
| Test execution | □ | □ | □ | □ | □ |
| Test interpretation | □ | □ | □ | □ | □ |
| Communication with other services | □ | □ | □ | □ | □ |
| Contacts with family for missing neonates/infants | □ | □ | □ | □ | □ |
| Data management | □ | □ | □ | □ | □ |
| Reporting | □ | □ | □ | □ | □ |
| Other, specify ______________________ | □ | □ | □ | □ | □ |

**MONITORING, VERIFYING AND REPORTING PHASE**

**Universality**

- Observation period: from _______ [MM/YYYY] to _______ [MM/YYYY]
- _______ [Number - #] newborns screened by 1 month of age out of _________ [#] newborns (____%);
- Reports from steps:

| **Step / Phase** | **Indicator** | **Higher risk**  **[#]** | **Low risk**  **[#]** |
| --- | --- | --- | --- |
| **1** | Tested | ______ | ______ |
|  | Referred/positive | ______ | ______ |
| **2** | Tested | ______ | ______ |
|  | Of which, number tested coming from home birth / missed or incomplete at the previous step | ______ | ______ |
|  | Referred/positive | ______ | ______ |
| **3** | Tested | ______ | ______ |
|  | Of which, number tested coming from home birth / missed or incomplete at the previous steps | ______ | ______ |
|  | Referred/positive | ______ | ______ |
| **4** | Tested | ______ | ______ |
|  | Of which, number tested coming from home birth / missed or incomplete at the previous steps | ______ | ______ |
|  | Referred/positive | ______ | ______ |
| **Full Audiological evaluation** | Total Tested | ______ | ______ |
|  | Of which, number of tested coming from home birth / missed or incomplete at the previous steps | ______ | ______ |

**Timely detection**

- ______ [#] Infants identified with HL by 3 months of age out of ______ [#] screened neonates (____%);

| - Full Audiological evaluation | Number of children assessed who falls within one of the following intervals |  |  |
| --- | --- | --- | --- |
|  | 0 - 10 db HL | ______ | ______ |
|  | 11 - 20 db HL | ______ | ______ |
|  | 21 - 30 db HL | ______ | ______ |
|  | 31 - 40 db HL | ______ | ______ |
|  | 41 - 50 db HL | ______ | ______ |
|  | 51 - 60 db HL | ______ | ______ |
|  | 61 - 70 db HL | ______ | ______ |
|  | 71 - 80 db HL | ______ | ______ |
|  | 81 - 90 db HL | ______ | ______ |
|  | 91+ db HL  TOT | ______  ______ | ______  ______ |

- Number of infants not having any screening exam (**not enrolled**) found to have hearing loss during Surveillance (excluded late onset cases): _______;
- Number of infants not completing the screening protocol (**lost to follow-up**) found to have hearing loss during the Surveillance (exclude late onset cases): _______;
- ______ [#] Infants found to have hearing loss during the Surveillance (excluded late onset cases) out of ______ [#] screened neonates not referred to full audiological evaluation as passed at the screening tests (____%) – *False negative*.

**Overreferral[[2]](#footnote-3)**

- Population without risk factors for HL: _______ [#] referred at discharge as eligible for further screening steps out of _______ [#] screened (____%)
- Population with risk factors for HL: _______ [#] referred at discharge as eligible for further screening steps out of _______ [#] screened (____%)
- Population without risk factors for HL: ________ [#] Newborns/Infants failed screening and rescreening before comprehensive audiological evaluation out of ______ [#] screened neonates (____%);
- Population with risk factors for HL: ________ [#] Newborns/Infants failed screening and rescreening before comprehensive audiological evaluation out of ______ [#] screened neonates (____%)

**References**

1. e.g. Slight, Mild, Moderate, Moderately severe, Severe, Profound [↑](#footnote-ref-2)
2. The False positive indicators are not reported as calculable from data in the two previous sections [↑](#footnote-ref-3)
